# Association between poor parent-daughter relationships and the risk of hyperglycemia in pregnancy: a hospital-based prospective cohort study in Japan

**DOI:** 10.1186/s12884-023-05535-3

**Published:** 2023-04-04

**Authors:** Yuto Maeda, Satomi Doi, Aya Isumi, Shuhei Terada, Junichi Sugawara, Kazuhisa Maeda, Shoji Satoh, Nobuaki Mitsuda, Takeo Fujiwara

**Affiliations:** 1 Department of Global Health Promotion, Tokyo Medical and Dental University, 1-5-45 Yushima, Bunkyo-ku, Tokyo, 113-8519 Japan; 2 Japan Society for the Promotion of Science, Tokyo, Japan; 3 Division of Feto-Maternal Medical Science, Department of Community Medical Support, Tohoku Medical Megabank Organization, Tohoku University, Sendai, Japan; 4 Department of Obstetrics and Gynecology, Shikoku Medical Centre for Children and Adults, Kagawa, Japan; 5 Perinatal Center, Oita Prefectural Hospital, Oita, Japan; 6 Department of Maternal Fetal Medicine, Osaka Women’s and Children’s Hospital, Osaka, Japan

**Keywords:** Adverse childhood experiences, Gestational diabetes, Hyperglycemia, Parent-daughter relationship

## Abstract

**Background:**

Adverse child experiences (ACEs), childhood maltreatment and household dysfunction, are risk factors of hyperglycemia in pregnancy (HIP), including diabetes before pregnancy, gestational diabetes (GDM), and overt diabetes in pregnancy, through increased risk of unhealthy behaviors, obesity, and stress response system dysfunction. While ACEs are often difficult to be assessed in hospital settings, parent-daughter relationship, that is, pregnant women’s relationship with their parents can be considered as a measurable maker for ACEs that may be associated with HIP. The purpose of this study is to examine the association between poor parent-daughter relationship and HIP.

**Methods:**

Hospital-based prospective cohort study was conducted in Japan (N = 6,264). Women visiting participating 58 facilities for delivery between April 2019 and March 2020 were included. Parent-daughter relationship was assessed by a questionnaire asking whether participants were satisfied with their relationship with their parents. HIP was diagnosed based on the criteria used in Japan. A multiple logistic regression model was applied to adjust for covariates.

**Results:**

Pregnant women who were not very satisfied and not satisfied at all with the relationship with their parents, and HIP were 343 (5.5%), 74 (1.2%), and 274 (4.4%), respectively. Pregnant women who were not very satisfied with their parent-daughter relationship showed a significant positive association with HIP in the crude model (odds ratio (OR): 1.71, 95% confidence interval (CI): 1.11–2.63). When stratified by psychiatric disease history, we found a significant positive association among those without psychiatric disease history (OR: 1.77, 95% CI: 1.11–2.84), but not among those with psychiatric disease history (OR: 0.61, 95% CI: 0.16–2.28).

**Conclusions:**

Poor parent-daughter relationship was associated with the risk of HIP among pregnant women without psychiatric disease history, suggesting that this simple question could be used to estimate the risk of HIP when it was challenging to inquire directly about ACEs. Further research is needed to elucidate the mechanism of the association.

**Supplementary Information:**

The online version contains supplementary material available at 10.1186/s12884-023-05535-3.

## Background

Hyperglycemia in pregnancy (HIP), including diabetes before pregnancy, gestational diabetes mellitus (GDM), and overt diabetes in pregnancy, is a common condition, estimated to be complicated with 15.8% of live births in the world [1]. Since treatment of HIP can reduce several morbidities, such as preeclampsia, macrosomic newborn, and shoulder dystocia, while the cost of treatment was only an increase in the number of prenatal visits [2], screening of HIP is now essential for the obstetrical practice.

Adverse childhood experiences (ACEs), including physical, psychological, and sexual abuse, neglect, and household dysfunction, have been well reported to influence adult health profoundly [3]. Several studies showed that ACEs impacted mental health and physical well-being, such as heart disease, stroke, and diabetes [3–5]. As pregnancy is a natural stress test for the future development of chronic disease, groups at high risk of diabetes, that is, women with multiple ACEs, are more likely to develop HIP. [6, 7]

Poor parent-daughter relationships can be one phenotype of ACEs, as poor parent-child relationships were also associated with youth smoking, unhealthy weight control behavior, obesity, and psychological distress in adulthood [8–10]. Thus, the pregnant women’s relationship with their parents may be associated with HIP risk. Moreover, assessing the parent-daughter relationship is more acceptable than assessing ACEs in a clinical setting and applicable to detecting high-risk groups of HIP [11].

When analyzing the association between poor parent-daughter relationships and HIP, the presence of psychiatric diseases should be considered because poor parent-child relationships are associated with psychiatric diseases [10, 12], and psychiatric diseases are known risk factors for diabetes or GDM [13]. Furthermore, several studies showed that psychiatric diseases increased HIP risk through obesity due to reduced frequency and intensity of exercise and side effects of psychotropic agents [13, 14]. Besides, a study on 6,317 pregnant women in Australia showed that women with three or more ACEs significantly had an increased risk of GDM only among those with psychiatric diseases [6]. This study suggested that psychiatric diseases need to be considered as mediators or effect modifiers. Thus, we should carefully consider the involvement of psychiatric diseases in analyzing the relationship between poor parent-daughter relationships and HIP risk. That is, we need further stratified analysis by the existence of psychiatric diseases.

Therefore, using a hospital-based sample, we attempted to clarify whether the parent-daughter relationship influenced the risk of HIP, with carefully considering the impact of the result by the presence of psychiatric diseases.

## Methods

### Participants

**Fig. 1:**
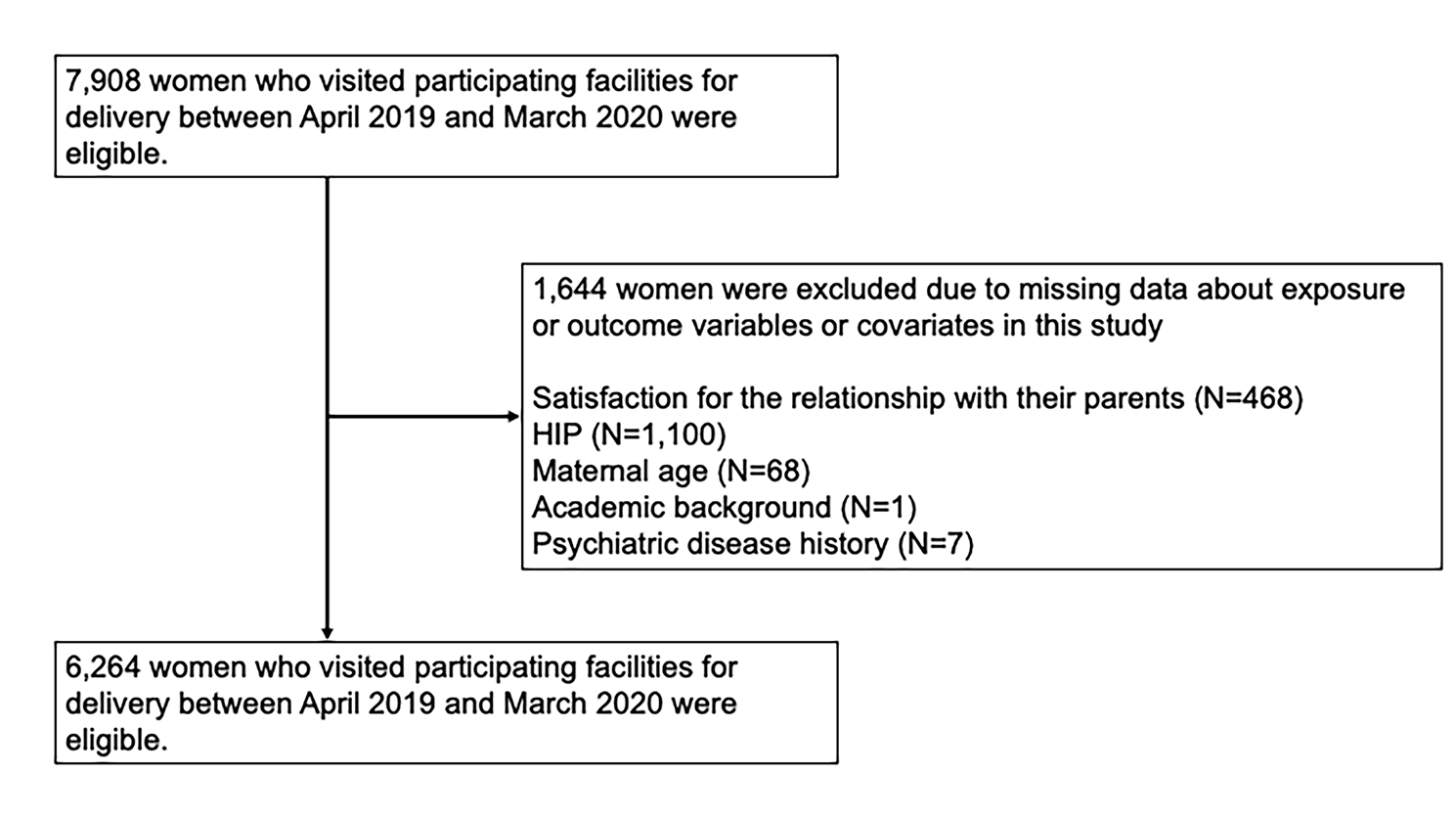
Flow diagram of participants included in the study

This study was a hospital-based prospective cohort study for the development of the Social Life Impact for Mother (SLIM) scale in the first trimester to identify mothers who need social support during postpartu[15]. Participants were recruited at obstetric hospitals in four prefectures. ((N = 6242) Miyagi, 14.7% Osaka, 37.0%, Kagawa, 6.4%, and Oita, 41.9%) These prefectures cover the eastern and western parts of Japan, and 58 hospitals out of 214 delivery facilities in this region participated in the study. These hospitals ranged from urban perinatal centers to regional obstetric care facilities; all women who visited participating facilities for delivery between April 2019 and March 2020 were included. Participants were asked to answer questionnaires on their first visit to the facilities. A questionnaire was distributed to 7,908 women. Written informed consent was obtained from all study participants. All women enrolled in the study were followed until one month postpartum. The exclusion criteria are as follows; missing data about exposure or outcome variables or covariates in this study (i.e., satisfaction for the relationship with their parents (N = 468), HIP (N = 1,100), maternal age (N = 68), academic background (N = 1), and psychiatric disease history (N = 7)). The final analytical sample for primary analysis included 6,264 women (Fig. 1). This study was conducted according to the guidelines of the Declaration of Helsinki and was approved by the institutional review boards of the core institution of four prefectures, that is, Osaka Women’s and Children’s Hospital (No.1125), Shikoku Medical Center for Children and Adults (H30-38), Oita Prefectural Hospital (30–70), and Tohoku University Hospital (2018-4-108).

### Measurement

The primary interest of outcome was the incidence of HIP, including diabetes before pregnancy, GDM, and overt diabetes in pregnancy. Information about HIP was collected by a questionnaire to medical institutions. In Japan, it is common to register information about preconception diabetes, GDM, and overt diabetes in pregnancy collectively, and the questionnaire used in this study also followed that practice. GDM was diagnosed when one or more values during the 75 g oral glucose tolerance test were greater than the cutoff levels: fasting plasma glucose ≥ 92 mg/dL, 1 h value ≥ 180 mg/dL, and 2 h value ≥ 153 mg/dL. When fasting blood sugar level is more than 126 mg/dl, and/or HbA1c level is more than 6.5% at their first visit, they are diagnosed with overt diabetes in pregnancy [16], and diabetes before pregnancy is diagnosed based on the criteria defined by the Japanese Association of Diabetes [17]. Women with all forms of diabetes described above were included in this study.

Pregnant women’s satisfaction with their relationship with their parents was asked by using the following question: “Are you satisfied with the relationship you have with your parents? Please circle each of the following: satisfied, not very satisfied, not satisfied at all.” Criterion validity was confirmed by comparing the question with social support status (whether there is someone you can talk to when you need help), based on a previous study showing that parent-daughter relationship determined social support in adulthoo[18]. When the answers of “not very satisfied” and “not satisfied at all” were considered abnormal, the sensitivity and specificity of identifying women without the support of their surroundings were confirmed to be 93.9% and 75%, respectively. In addition, the predictive validity of the questionnaire was confirmed in another study to examine the association between parent-daughter relationship trajectories and postpartum depressio[19].

As covariates, maternal characteristics, including maternal age [20], psychiatric disease histories [13] (“No,” “Yes (past),” or “Yes (current)”), and academic background [21] (“high school or more,” “retirement from high school,” “junior high school”) were assessed with a questionnaire at their first visits.

### Analysis

We used the chi-square test for discrete and Student’s t-test for continuous variables to compare maternal demographics stratified by status of HIP. The normality of continuous variables was checked by seeing the distribution of the data with a histogram. A multiple logistic regression model was applied to examine the association between parent-daughter relationship and diabetes or gestational diabetes. In this analysis, we considered maternal age [20], psychiatric disease histories [13], and academic background [21] as covariates. In addition, we performed multivariate logistic regression analysis stratified by psychiatric disease history, considering psychiatric disease history might be an effect modifier. Further, we used causal-mediation analysis to examine mediation effect of psychiatric disease histories [13, 14]. In addition to the primary analysis, we conducted a sensitivity analysis for the association between parent-daughter relationship and HIP on the sub-set of all recruited participants after replacing missing or unreliable data using 200 sets of multiple imputations. We performed all statistical analyses using the statistical software package, Stata SE 15 (STATA Corp, College Station, TX), and considered p < 0.05 statistically significant.

## Results

Maternal backgrounds stratified by satisfaction for relationships with parents were shown in Table 1. 4.4% (274/6264) of women were complicated with HIP. Women who were satisfied with their relationship with patents as “not very satisfied” were 5.5% (343/6,264), and those who were “not satisfied at all” were 1.2% (74/6,264). Women with HIP were more likely to be older, have psychiatric disease histories, and not to be satisfied with their relationships with their parents. Women in Kagawa prefecture were more likely to have HIP than in other prefectures. Other background characteristics were not significantly different between the two groups.

**Table 1:** Characteristics of the sample by satisfaction for relationship with parents

Variables	Total (N = 6,264)	Satisfaction for relationship with parents			
		Satisfied (N = 5,847)	Not very satisfied (N = 343)	Not satisfied at all (N = 74)	p-value
	Mean (SD) or N (%)				
Gestational age at delivery (weeks) (N = 5124)	38.8 (2.4)	38.8 (2.4)	38.8 (1.5)	38.1 (4.7)	0.18
Nulliparity (N = 5234)	2201 (42.1)	2075 (42.4)	101 (36.9)	25 (40.3)	0.19
**Maternal age at delivery (year)**					0.19
15–19	61 (1.0)	58 (1.0)	2 (0.6)	1 (1.4)	
20–24	579 (9.2)	534 (9.1)	36 (10.5)	9 (12.2)	
25–29	1713 (27.4)	1621 (27.7)	73 (21.3)	19 (25.7)	
30–34	2206 (35.2)	2056 (35.2)	127 (37.0)	23 (31.1)	
35–39	1375 (22.0)	1276 (21.8)	82 (23.9)	17 (23.0)	
40–44	320 (5.1)	293 (5.0)	23 (6.7)	4 (5.4)	
45–49	9 (0.1)	8 (0.1)	0 (0.0)	1 (1.4)	
50–54	1 (0.02)	1 (0.02)	0 (0.0)	0 (0.0)	
**Prefecture of the institutions**					0.06
Osaka	2315 (37.0)	2182 (37.3)	111 (32.4)	22 (29.7)	
Miyagi	923 (14.7)	854 (14.6)	51 (14.9)	18 (24.3)	
Kagawa	399 (6.4)	375 (6.4)	18 (5.3)	6 (8.1)	
Oita	2627 (41.9)	2436 (41.7)	163 (47.5)	28 (37.8)	
**Academic background**					< 0.01
Highschool graduate or more	5980 (95.4)	5605 (95.9)	312 (91.0)	63 (85.1)	
High school dropout	174 (2.8)	153 (2.6)	16 (4.7)	5 (6.8)	
Junior high school graduate	110 (1.8)	89 (1.5)	15 (4.4)	6 (8.1)	
Psychiatric disease history	350 (5.6)	276 (4.7)	54 (15.7)	20 (27.0)	< 0.01
Hyperglycemia in pregnancy	274 (4.4)	247 (4.2)	24 (7.0)	3 (4.1)	0.05
**Fetal birthweight (g) (N = 5207)**					0.10
<1500	29 (0.6)	25 (0.5)	4 (1.5)	0 (0.0)	
1500〜2500	427 (8.2)	397 (8.2)	21 (7.7)	9 (14.3)	
>2500	4751 (91.2)	4450 (91.3)	247 (90.8)	54 (85.7)	

Associations between HIP and satisfaction for relationships with parents or covariates were shown in Table 2. Those who were not so much satisfied with their relationship with their parents were significantly at high risk of HIP in a crude model of logistic regression analysis (odds ratio [OR]: 1.71, 95% confidence interval [CI]: 1.11–2.63), but when we add a history of maternal psychiatric disease, it became non-significant in multiple logistic regression analysis (OR: 1.53, 95%CI: 0.98–2.39). In contrast, those who were not satisfied with their relationships with their parents at all did not have a high risk of HIP in the crude model (OR: 0.96, 95%CI: 0.30–3.06) and in the adjusted model (OR: 0.78, 95%CI: 0.24–2.55).

**Table 2:** Association between hyperglycemia in pregnancy and satisfaction for relationship with parents or covariates

Variables	Crude OR^a^ [95% CI]^b^	Adjusted OR [95% CI]^c^	p-value
**Satisfaction for relationship with parents**			
Satisfied	reference	reference	reference
Not very satisfied	1.71 (1.11–2.63)	1.53 (0.98–2.39)	0.06
Not satisfied at all	0.96 (0.30–3.06)	0.78 (0.24–2.55)	0.68
**Psychiatric disease history**	1.75 (1.14–2.68)	1.58 (1.02–2.44)	0.04
**Maternal age at delivery (year)**			
15–19	1.56 (0.55–4.39)	1.22 (0.42–3.59)	0.71
20–24	0.27 (0.13–0.59)	0.26 (0.12–0.56)	< 0.01
25–29	0.70 (0.49–0.98)	0.69 (0.49–0.97)	0.04
30–34	reference	reference	reference
35–39	1.61 (1.20–2.16)	1.60 (1.19–2.15)	< 0.01
40–44	1.64 (1.02–2.65)	1.61 (1.00-2.61)	0.05
45–49	2.78 (0.34–22.43)	3.09 (0.38–25.04)	0.29
50–54	-	-	-
**Academic background**			
University or more	reference	reference	reference
High school graduate	1.66 (0.91–3.03)	1.98 (1.06–3.69)	0.03
Junior high school graduate	1.53 (0.70–3.31)	1.52 (0.68–3.39)	0.30

Note:

a: OR = Odds Ratio b: CI = Confidence Interval

c: Covariates included maternal age, academic background, psychiatric disease history

Association between HIP and satisfaction for the relationship with parents stratified by psychiatric disease history was shown in Table 3. Among the group without psychiatric disease histories, women who were not very satisfied with their relationship with their parents showed a significant increase in the risk of HIP (OR: 1.77, 95%CI: 1.11–2.84). In contrast, among the group with psychiatric disease histories, satisfaction for the relationship with parents was not associated with HIP (OR: 0.61, 95%CI: 0.16–2.28).

**Table 3:** Association between hyperglycemia in pregnancy and satisfaction for relationship with mothers stratified by psychiatric disease history

Variables	Crude OR^a^ [95% CI]^b^	Adjusted OR [95% CI]^c^
**Psychiatric disease history (-)**		
Satisfaction for relationship with parents		
Satisfied	reference	reference
Not very satisfied	1.85 (1.17–2.95)	1.77 (1.11–2.84)
Not satisfied at all	0.91 (0.22–3.76)	0.85 (0.20–3.56)
**Psychiatric disease history (+)**		
Satisfaction for relationship with parents		
Satisfied	reference	reference
Not very satisfied	0.71 (0.21–2.48)	0.61 (0.16–2.28)
Not satisfied at all	0.64 (0.08–5.01)	0.60 (0.07–4.93)

Note:

a: OR = Odds Ratio b: CI = Confidence Interval

c: Covariates included maternal age, academic background

Effect decomposition of the total effect of parent-daughter relationship due to psychiatric disease history on HIP was shown in Table S1. The association between the relationship with parents and HIP was not mediated by psychiatric disease history (natural indirect effect; OR: 1.00, 95%CI: 0.93–1.07). Multiple imputation analysis showed a similar tendency to the primary analysis (Table S2, S3).

## Discussion

To the best of our knowledge, this is the first study to show the association between poor relationships with parents and the risk of HIP among pregnant women, using a prospective hospital-based study in Japan. Furthermore, this association differed by the psychiatric disease history and was significant only among the population without a psychiatric disease history, suggesting that the association was modified by psychiatric disease history.

The result of our study was consistent with previous studies about ACEs and HIP. Although we did not directly ask whether participants had a history of child abuse and neglect due to social barriers in a clinical setting in Japan, their poor relationships with parents may indicate a history of childhood maltreatment (abuse and neglect), as shown in a previous meta-analysis [22]. Thus, we used the question of whether women are satisfied with their parents as a proxy of the history of child maltreatment in our study, which was feasible in clinical practice. It is crucial that parent-daughter relationship was identified as a factor in identifying patients at high risk for HIP, one of the important pregnancy complications, because asking about childhood maltreatment history is often burdensome for pregnant women and can be a triggered trauma, which may hamper the patient-physician relationship. In contrast, the other two previous studies on 2,319 Hispanic or Latino women and on 1,274 women in the United States reported that ACE scores were not associated with GDM. This discrepancy could partly be explained by the difference in race or ethnicity, the distribution of ACEs among the population in each study, or the reliability of the ACE questionnaire in these previous studies, i.e., patients may reluctant to report their ACE if asked directly. Further studies are warranted to clarify the relationship between parent-daughter relationship and ACEs, and to investigate their association with HIP.

The possible mechanism by which the parent-daughter relationship may increase the risk of HIP is through an increase in youth smoking [8] and unhealthy weight control behaviors [9]. A previous study by telephone interview on 428 youth-parent pairs in the United States showed that among youth whose parents did not smoke, those who reported poor parent-child connectedness were twice as likely to have ever smoked as those who reported high connectedness [8]. Another study on 4,746 students in public schools in the United States showed that students who reported poor relationships with their mothers had a higher prevalence of unhealthy weight management behaviors than those who reported that they felt their mothers cared about them [9]. In addition, a poor parent-daughter relationship can increase the frequency of obesity, which is an essential risk for HIP. Previous studies have suggested that poor parent-child relationships caused obesity in children through their effects on stress-induced hypothalamic-pituitary-adrenal (HPA) axis signaling neuroendocrine changes [23] and impairment of children’s capacity for self-regulation [24].

This study showed that the groups with lower satisfaction for parent-daughter relationships were associated with HIP, whereas the group with no satisfaction for parent-daughter relationships was not. The possible reason for this result may be due to the child welfare system in Japan. Those who were not satisfied with parent-daughter relationship at all were at higher risk for multiple ACEs, including child maltreatment, and thus they may have been supported by the child welfare system from childhood, paradoxically attenuating the effect of ACEs on HIP [25].

In the current study, the pregnant women’s relationship with parents was significantly associated with HIP risk in women without psychiatric diseases but not in women with psychiatric diseases. Interestingly, this was inconsistent with the previous study. The study on 6,317 women in Australia suggested that three or more ACEs and individual subcategories of physical abuse significantly increased the risk of GDM among women with preconception depressive symptoms but not among women without preconception depressive symptoms [6]. This study explained that preconception depressive symptoms were a mediating factor in the relationships between ACEs and GDM. However, it was possible that preconception depressive symptoms were an effect modifier, as shown in the current study. This effect can partly be explained by public health services during pregnancy in Japan. Pregnant women with psychiatric diseases are identified as parents with difficulties raising their children to prevent child abuse by public health nurses in each municipality [26]. However, pregnant women with poor parent-daughter relationships, one of the phenotypes of ACEs, and without psychiatric diseases are not routinely supported by public health nurses in Japan. Thus, women without psychiatric diseases are less likely to receive public health services and appropriate treatment to reduce the effects of poor parent-daughter relationships. As a result, women without psychiatric diseases may develop HIP due to the accumulated effects of the poor parent-daughter relationship after they suffer from the burden of pregnancy. Further studies to clarify the effect of psychiatric disease on the association between poor parent-daughter relationships and HIP are warranted.

The present study had several limitations. First, the samples used in this study were extracted from four prefectures, and Japan has 47 prefectures, which posed a problem in terms of generalizability for application to the whole population in Japan. Second, since HIP, the primary interest of outcome in this study, was collected as one category, we could not differentiate diabetes before pregnancy, GDM, and overt diabetes in pregnancy. However, since the underlying mechanism of the effect of parent-daughter relationships on HIP were mainly common regardless of the subcategories of HIP, we considered all the subcategories of HIP as one category. Third, we did not consider some potential covariates, such as body mass index, smoking status, diet, blood pressure, and gestational weight gain, which could affect the risk of HIP. However, parent-daughter relationships were upstream of the pathway, that is, chronologically before maternal weight, blood pressure, and diet, and thus might be mediating factors of the association between parent-daughter relationships and HIP, as previous studies suggested. Therefore, we did not include those covariates in the analysi[20]. Fourth, since the questionnaire in this study was self-administered, there may be response bias.

## Conclusions

In conclusion, poor relationships with their parents assessed at the first pregnancy visit were significantly associated with the risk of HIP among pregnant women with no psychiatric disease history. The results suggested that this simple question could be used to estimate the risk of HIP in clinical settings where it was challenging to inquire directly about childhood maltreatment history. Further studies, including various confounding factors, such as BMI, GWG, and smoking status, were warranted.

## Electronic supplementary material

Below is the link to the electronic supplementary material.

**Additional file 1**. Supplementary tables

## Data Availability

The datasets used and/or analysed during the current study are available from the corresponding author on reasonable request.

## Abbreviations

ACEs: Adverse childhood experiences
HIP: Hyperglycemia in pregnancy
GDM: gestational diabetes mellitus
OR: odds ratio
CI: confidence interval
BMI: body mass index
GWG: gestational weight gain
