# Supplementary material for: Association between poor parent-daughter relationships and the risk of hyperglycemia in pregnancy: a hospital-based prospective cohort study in Japan
Source: BMC Pregnancy Childbirth. 2023 Apr 4;23:227. doi: 10.1186/s12884-023-05535-3 (PMC10071734; doi:10.1186/s12884-023-05535-3)
Supplement: Supplementary file 1 — Additional file 1. Supplementary tables [file 12884_2023_5535_MOESM1_ESM.pdf]

Supplementary Table1 Effect Decomposition of Total Effect of parent-child relationship due to psychiatric disease history on hyperglycemia in pregnancy

	OR [95% CI][a]	p-value
mte	1.29 (0.92-1.82)	0.14
nde	1.29 (0.91-1.84)	0.16
nie	1.00 (0.93-1.07)	0.98

Note:
OR=Odds Ratio, CI=Confidence Interval,
mte=marginal total effect, nde=natural direct effect,
nie=natural indirect effect
a: Covariates included maternal age, academic background

Supplementary Table 2 Multiple imputation analysis for the association between hyperglycemia in pregnancy and satisfaction for relationship with parents or covariates

	Crude OR[a] [95% CI][b]	Adjusted OR [95% CI][c]	p-value
**Satisfaction for relationship with parents**			
Satisfied	reference	reference	reference
Not very satisfied	1.66 (1.07-2.57)	1.50 (0.96-2.34)	0.08
Not satisfied at all	1.29 (0.47-3.52)	1.02 (0.37-2.82)	0.97
**Psychiatric disease history**	1.74 (1.13-2.65)	1.54 (0.99-2.37)	0.05
**Maternal age at delivery (year)**			
15-19	reference	reference	reference
20-24	0.17 (0.050-0.61)	0.21 (0.059-0.76)	0.02
25-29	0.45 (0.16-1.28)	0.56 (0.19-1.67)	0.30
30-34	0.64 (0.23-1.80)	0.82 (0.28-2.40)	0.71
35-39	1.03 (0.37-2.91)	1.31 (0.45-3.85)	0.62
40-44	1.05 (0.35-3.17)	1.32 (0.45-3.85)	0.64
45-49	1.78 (0.18-18.00)	2.53 (0.24-26.15)	0.44
50-54	-	-	-
**Academic background**			
University or more	reference	reference	reference
High school graduate	1.66 (0.92-3.00)	2.04 (1.11-3.73)	0.02
Junior high school graduate	1.52 (0.69-3.34)	1.60 (0.72-3.57)	0.25

Note:

a: OR=Odds Ratio b: CI=Confidence Interval

c: Covariates included maternal age, academic background, psychiatric disease history

Supplementary Table 3 Multiple imputation analysis for associations between hyperglycemia in pregnancy and satisfaction for relationship with mothers stratified by psychiatric disease history

	Crude OR[a] [95% CI][b]	Adjusted OR [95% CI][c]
**Psychiatric disease history (-)**		
**satisfaction for relationship with parents**		
Satisfied	reference	reference
Not very satisfied	1.79 (1.13-2.85)	1.73 (1.09-2.77)
Not satisfied at all	1.29 (0.40-4.14)	1.17 (0.36-3.81)
**Psychiatric disease history (+)**		
**satisfaction for relationship with parents**		
Satisfied	reference	reference
Not very satisfied	0.77 (0.23-2.58)	0.71 (0.20-2.49)
Not satisfied at all	0.78 (0.10-5.77)	0.59 (0.076-4.60)

Note:

a: OR=Odds Ratio b: CI=Confidence Interval

c: Covariates included maternal age, academic background
